# Bacteria profiles and antimicrobial susceptibility pattern of isolates from beds and door handles of hospital wards in Tiko Health District, Cameroon

**DOI:** 10.11604/pamj.2024.49.85.41817

**Published:** 2024-11-21

**Authors:** Njeodo Njongang Vigny, Binwie Fanuella Shu

**Affiliations:** 1 Department of Medical Laboratory Science, Faculty of Health Sciences, University of Buea, Buea, Cameroon,; 2 Department of Applied Science, School of Engineering and Applied Sciences, *Institut Universitaire de la Côte*, Douala, Cameroon,; 3 Department of Medical Laboratory Science, School of Medical and Biomedical Sciences, Maflekumen Higher Institute of Health Sciences Tiko, Tiko, Cameroon

**Keywords:** Antimicrobial susceptibility patterns, bacterial isolates, door handles, beds, hospital wards, Tiko Health District

## Abstract

**Introduction:**

in low- and middle-income countries, hospital surfaces contaminated with bacteria, namely beds and door handles in hospital wards, are a major source of nosocomial infections. We sought to evaluate bacterial isolates from beds and door handles of hospital wards and ascertain their antibiotic susceptibility patterns in Tiko Health District (THD), Cameroon.

**Methods:**

using a multistage sampling technique, this hospital-based cross-sectional study included 40 beds and 20 door handles in THD. Gram staining methods, biochemical reactions, and features of bacterial colonies were used to identify bacterial isolates. A frequency table and bar charts were used to display the data.

**Results:**

Bacillus spp., Clostridium perfringens, Klebsiella pneumoniae, Clostridium spp., and Staphylococcus aureus were identified. Patient beds were mainly contaminated with S. aureus (42.5%, 17/40). However, C. perfringens (35%, 7/20) was the most common isolate from door handles. S. aureus was resistant to bacitracin (100%, 21/21) but sensitive to gentamycin (95.2%, 20/21) and azithromycin (95.2%, 20/21). While C. perfringes was resistant to bacitracin (100%, 8/8), it was sensitive to gentamycin (75%, 6/8) and chloramphenicol (75%, 6/8).

**Conclusion:**

beds and door handle harbour largely S. aureus and C. perfringes, respectively. High sensitivity to gentamycin and resistance to bacitracin were observed in S. aureus and C. perfringes, respectively. Good and regular hand hygiene and the cleaning and disinfecting of door knobs and hospital beds should be practiced. Hospitals should fully adopt food safety protocols to prevent or control food poisoning effectively.

## Introduction

Hospital-acquired infections (HAIs), also known as nosocomial infections, are illnesses that occur 48 hours following a hospital visit or during hospitalization [1]. Bacteria, viruses, fungi, and parasites are linked to HAIs. In addition, *Staphylococcus aureus, Enterobacteria, Pseudomonas aeruginosa, Klebsiella pneumoniae*, and *Clostridium difficile* are the primary bacterial agents. Aspergillus and candida are also related to HAIs, while rotavirus, hepatitis B and C viruses, and influenza virus are viruses that cause HAIs [2,3]. A diverse range of microorganisms can be found in hospital environments, with a few pathogenic bacterial strains frequently colonizing regularly touched areas such as door knobs, beds, bed rails, trays, and tables among others [4]. According to research done in Douala, Garoua, and Dschang, surgical site infection is the most common of the HAIs, which also include respiratory, gastrointestinal, bloodstream, and urinary tract infections. In addition, the surgical ward had a higher prevalence of skin and soft tissue site infections than urinary and bedsore infections [5,6]. Because of patients' irrational and excessive use of antibiotics, bacteria that cause HAIs most frequently have become resistant to them [7].

According to a meta-analysis, the prevalence of HAIs is 0.14% globally and is increasing by 0.06% a year [8]. According to the same study, *E. coli* had the highest infection rate among patients when compared to Coagulase-negative Staphylococci (CoNS), *Staphylococcus spp*., and *P. aeruginosa*. Additionally, the global prevalence of HAIs was higher in men than in women [8]. The Americas and the West Pacific had the lowest rates of HAIs, while Africa had the greatest rate [8]. Furthermore, the prevalence of HAIs in Africa (12.76%) is more than twice as high as that seen in developed nations [9]. Based on positive culture results for HAIs in records from the microbiology unit, gram-negative bacteria (*K. pneumoniae, Escherichia coli, P. aeruginosa, Acinetobacter baumannii*, and *Citrobacter*) were the most prevalent microorganisms in this region and accounted for 40%-100% of infections [9].

Research carried out in Nepal, Morocco, Ghana, Algeria, and the south, southwest, and northwest Ethiopia has revealed *S. aureus* to be the most common bacterial pathogen found on hospital ward door handles [10-16]. Other bacterial isolates have also been discovered in several of these investigations, including *P. aeruginosa, Enterobacter, CoNS, Staphylococcus saprophticus, Klebsiella* spp., and *Acinetobacter baumannii* [11,13,14,16]. On hospital ward beds, *S. aureus* and CoNS have frequently been found [13-16]. Conversely, there have not been many reports of Acinetobacter, *K. rhnioscleromates*, Enterobacter, and *E. coli* [14,16,17]. The most effective antibiotics against *S. aureus*, according to recent data from Southern Ethiopia, Nepal, Morocco, and Ghana, are ampicillin, gentamycin, levofloxacin, and ciprofloxacin, in that order [10-12,16].

At the University Teaching Hospital in Yaoundé, Cameroon, the cumulative incidence rate (19.21%) and death rate (28%) of HAIs are high [1]. Furthermore, 44.7% (25/85) of the hospital's wards had the greatest rate of bacterial contamination [18]. Data on microorganisms found on hospital wards' beds and door handles, as well as their patterns of antibiotic susceptibility, are scarce in Cameroon. Thus, the purpose of this study was to evaluate bacterial isolates from beds and door handles in hospital wards and determine their antibiotic susceptibility patterns in the Tiko health district of Cameroon.

## Methods

**Study duration, setting, and sampling technique:** this was a cross-sectional study that was conducted in Tiko Health District, for over six months, from November 2021 to April 2022, precisely at Regina Pacis Hospital, Private Medical Insurance (PMI) Hospital, COMWEL Clinic, and Tiko District Hospital. These four health facilities belong to the peripheral level in the structure of the health system in Cameroon. The Tiko Health District is one of the 18 health districts in the Southwest Region of Cameroon. The majority of the people who visit these hospitals are farmers and plantation workers [19]. The health district is found in the Southwest Region of Cameroon and has about 334,647 inhabitants distributed in eight health areas with a total surface area of 4840 km^2^ [20]. It has mean daily temperatures ranging from 28 to 33°C, which accounts for the dry nature of the study site [21]. It also has three urban settlements, namely Tiko, Likomba, and Mutengene, along with the following boundaries: Buea Health District (North), Bonaberi Health District (South), Dibombari Health District (East), and Limbe Health District (West), characterized by activities such as trading, farming, and fishing [20]. Maflekumen Teaching Laboratory is a site reserved for daily practicals on campus at the Maflekumen Higher Institute of Health Sciences. It has microscopes, an incubator, and a staining section with several cupboards in which stains, petri dishes, and slides are stored.

A total of 20 door handles and 40 beds were selected in the Tiko Health District of Cameroon using a multistage sampling technique. In the first stage, four health areas were randomly selected from the 8 health areas (Mutengene, Holforth, Kange, Tiko Town, Likomba, Misselele, Mondoni, and Mudeka) in the Tiko Health District. These selected health areas were Likomba, Tiko Town, Mutengene, and Holforth. In the next stage, a list of health facilities was compiled for each health area. The distribution of health facilities in the health areas was: Tiko town (COMWEL clinic), Holforth (PMI hospital), Mutengene (Baptist hospital, Atlantic hospital, Regina Pacis, and Centralized Medical hospital), and Likomba (Central clinic, Cottage hospital, Tiko district hospital, and Sone clinic). In stage 3, a hospital was randomly selected from each of the four health areas, resulting in the following facilities: Regina Pacis Hospital, PMI Hospital, COMWEL Clinic, and Tiko District Hospital. Five door handles and 10 beds were randomly selected from every hospital in the final stage.

**Inclusion and exclusion criteria:** beds and door handles of wards in Regina Pacis Hospital, PMI Hospital, COMWEL Clinic, and Tiko District Hospital were included. Nonetheless, beds and door handles outside the wards were excluded.

### Sample collection and processing

**Sample collection:** the surfaces of 40 beds and 20 door handles in the hospital wards were swabbed using sterile swab sticks moistened with 0.9% w/v physiologic saline. The samples (15 each from four hospitals) were collected using swab sticks, which were immediately put back in their sterile plastic tubes after collection. Face masks, gloves, and laboratory coats were worn to ensure the sterility of the samples. The tubes were finally transported in zip-lock polythene bags within 20-30 minutes to the Maflekumen Teaching Laboratory for processing.

### Sample processing

**Sample culture:** the swabbed samples were inoculated on CLED agar, blood agar, and MacConkey agar. Each agar was prepared by weighing it in powder form using an electronic balance and dissolving it in a measurable amount of distilled water, following the manufacturer´s instructions. During the preparation of the culture media, quality control was maintained by autoclaving the culture media to ensure sterility. It was later incubated aerobically at 37°C for 24 hours. Culture plates were inspected to identify visible bacteria colonies. Presumptive identifications of bacteria were done based on colony characteristics and Gram reactions. Confirmatory tests were done based on the enzymatic and biochemical reactions of the organisms using various reagents and enzymes. The pH of the enzymes and biochemical reagents was tested using pH papers to ensure quality control. They include the Catalase test (exclusively used to identify *S. aureus* and *Bacillus spp*.), the Nagler reaction (biochemical test for *C. perfringens*), the analytical profile index (biochemical test for *Enterobacteria spp*.), and the Indole test (commonly used to detect enterobacteria).

**Antimicrobial susceptibility testing:** antimicrobial susceptibility testing was performed for each bacterial isolate on nutrient agar using the Kirby-Bauer disk diffusion technique. Furthermore, three to five selected colonies of a pure culture of bacteria were transferred to a tube containing 5 ml of sterile normal saline and mixed gently to form a homogenous suspension until the turbidity of the suspension was adjusted to 0.5 McFarland [22]. A sterile cotton swab was used to remove the excess suspension by rotating the swab against the surface of the tube. The swab was then used to distribute the bacteria evenly over the surface of the nutrient agar. The inoculated plates were left at room temperature to dry for 3-5 minutes, and a set of antibiotic discs was placed on the inoculated plates using sterile forceps. The plates were allowed to stand for 30 minutes and later incubated at 35°C for 16 to 18 hours. Moreover, the observed zones of inhibition around the antibiotic discs were compared with the standard zones of inhibition for each bacterial isolate. If an observed zone was equal to or greater than the standard zone, the bacterial isolate was considered sensitive to a specific antibiotic. However, a bacterial isolate was described as resistant if otherwise. Each diameter of the observed zone of inhibition for a specific disc was measured using a ruler in centimeters [22].

**Data analysis:** data were entered into Microsoft Excel and exported into SPSS (Statistical Package for Social Sciences) version 22 for analysis. Qualitative data were presented on bar charts, while quantitative data were summarized and presented in a table.

**Ethical approval statement:** an approval letter to conduct this study was obtained from Maflekumen Higher Institute of Health Sciences Tiko. In addition, administrative authorizations were obtained from the district medical officer in Tiko and a director from each of the four hospitals.

## Results

**Figure 1:**
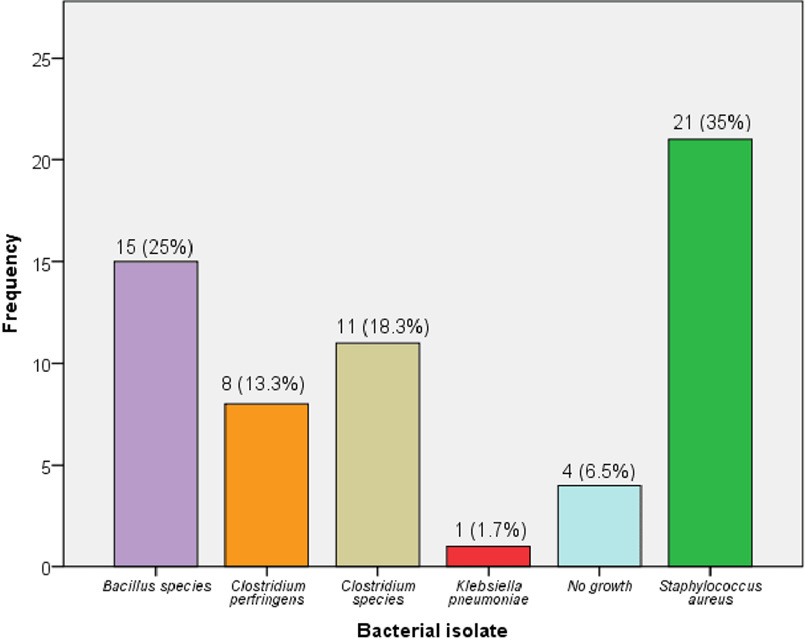
frequencies of bacterial isolates from beds and door handles of hospital wards in Tiko health district

**Frequencies of bacterial isolates from beds and door handles of wards in Tiko Health District:** the Tiko health District's hospital wards' beds and door knobs were found to harbour *S. aureus, C. perfringens, K. pneumoniae, Clostridium spp*., and *Bacillus spp*. Figure 1 illustrates that of the 60 bacterial isolates (found on hospital beds and door knobs in the Tiko Health District), the most common species were *Bacillus spp*.15 (25%) and *Staphylococcus aureus* 21 (35%).

**Figure 2:**
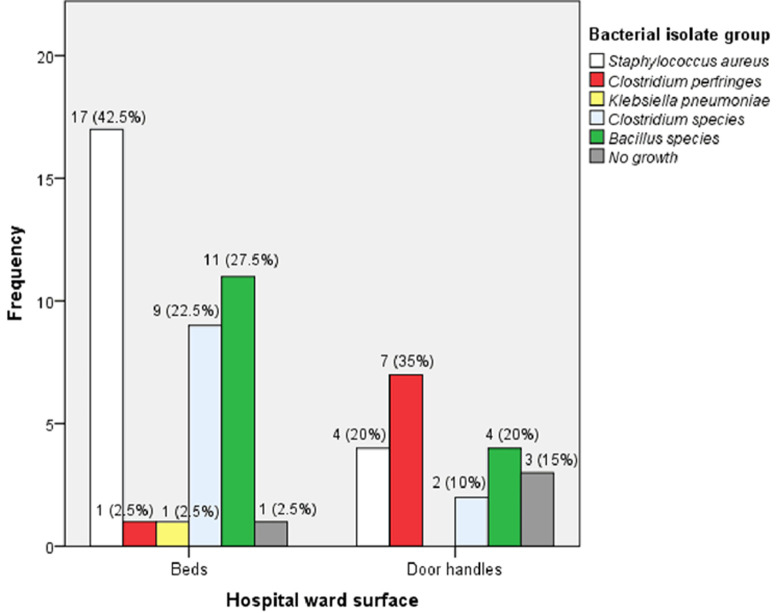
frequencies of bacterial isolates from hospitals based on ward surface

**Frequencies of bacterial isolates from hospital wards by beds and door handles:** compared to other bacterial isolates, *S. aureus* (42.5%, 17/40) and *Bacillus spp*. (27.5%, 11/40) were identified from ward beds more frequently. But the most frequent bacterial isolate from ward door handles was *C. perfringens* (35%, 7/20) (Figure 2).

**Figure 3:**
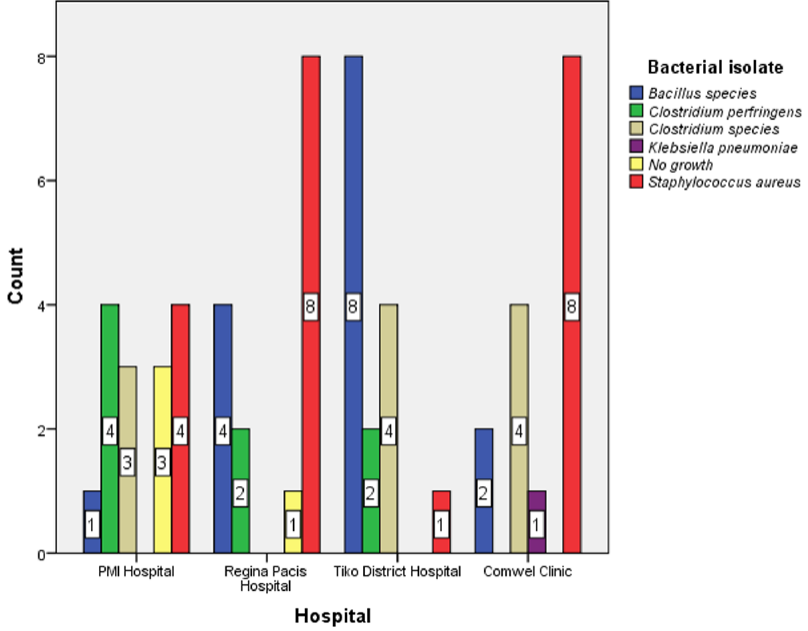
frequencies of bacterial isolates from wards’ beds and door handles based on hospital

**Frequencies of bacterial isolates from beds and door handles of wards by hospital:** Regina Pacis Hospital had a 53.3% prevalence of *S. aureus* on beds and door handles (8/15) and the COMWEL clinic had a similar prevalence (53.3%, 8/15). However, as Figure 3 illustrates, *Bacillus spp*. was isolated in Tiko District Hospital the most frequently (53.3%, 8/15).

Antimicrobial susceptibility patterns of *Staphylococcus aureus, Clostridium perfringes, Bacillus species*, and *Klebsiella pneumoniae: S. aureus* (a total of 21 isolates) were sensitive to gentamycin 20 (95.2%), azithromycin 20 (95.2%), ampicillin 9 (42.9%), and chloramphenicol 8 (38.1%). Additionally, as indicated in Table 1, *Bacillus spp*. (a total of 15 isolates) exhibited susceptibility to gentamycin 13 (86.7%) and chloramphenicol 4 (26.7%). Table 1 displays multidrug resistance in *S. aureus* against bacitracin 21 (100%), and tetracycline 12 (57.1%), while *Bacillus spp*. displayed multidrug resistance against bacitracin 15 (100%), cephalothin 10 (66.7%), and tetracycline 1 (6.6%).

**Table 1:** antimicrobial susceptibility pattern of bacteria isolated from hospital ward beds and door handles in the Tiko health district

Bacterial species (n)	Pattern	Antimicrobial susceptibility, no (%)							
		C	CIP	GN	TET	AZI	BAC	CEP	AMP
Staphylococcus aureus (21)	S	8 (38.1)	-	20 (95.2)	-	20 (95.2)	-	-	9 (42.9)
	I	-	-	-	8 (38.1)	-	-	1 (4.8)	-
	S^R^	5 (23.8)	9 (42.9)	1 (4.8)	-	-	-	-	-
	R	-	-	-	12 (57.1)	-	21 (100)	-	-
Clostridium perfringes (8)	S	6 (75)	2 (25)	6 (75)	-	4 (50)	-	-	2 (25)
	I	-	-	-	6 (75)	-	-	-	-
	S^R^	2 (25)	-	2 (25)	-	2 (25)	-	-	6 (75)
	R	-	-	-	-	-	8 (100)	2 (25)	-
Bacillus species (15)	S	4 (26.7)	-	13 (86.7)	-	-	-	-	10 (66.7)
	I	10 (66.7)	10 (66.7)	-	4 (26.7)	4 (26.7)	-	-	-
	S^R^	1 (6.6)	-	2 (13.3)	-	1 (6.6)	-	-	-
	R	-	-	-	1 (6.6)	-	15 (100)	10 (66.7)	-
Klebsiella pneumoniae (1)	S	-	-	1 (100)	-	-	-	-	1 (100)
	I	-	-	-	-	1 (100)	1 (100)	1 (100)	-
	S^R^	1 (100)	-	-	-	-	-	-	-
	R	-	-	-	-	-	-	-	-

S: sensitive; I: intermediate; S^R^: resistant mutant; R: resistant; NA: not applicable; C: chloramphenicol; CIP: ciprofloxacin; GN: gentamycin; TET: tetracyline; AZI: azithromycin; AMP: ampicillin; BAC: bacitracin; CEP: cephalothin

## Discussion

We assessed bacterial isolates from beds and door handles of hospital wards in Tiko Health District and determined their antimicrobial susceptibility patterns. The following isolates: *S. aureus, C. perfringens, K. pneumoniae, Clostridium spp*., and *Bacillus spp*. were found in the Tiko Health District. Our finding showed that *S. aureus* 35% (21/60) and *Bacillus spp*. 25% (15/60) were the most dominant bacterial pathogens on beds and door handles of hospital wards. *S. aureus* (42.5%, 17/40) was the predominant contaminant on beds. On the contrary, *C. perfringens* (35% (7/20) was the most frequent bacterial isolate from door handles. In addition, *S. aureus* (n = 21) was most sensitive to gentamycin 20 (95.2%) and azithromycin 20 (95.2%), followed by ampicillin 9 (42.9%) and chloramphenicol 8 (38.1%). In contrast, the sensitivity rates of *C. perfringes* (n = 8) were 6 (75%) for gentamycin, 6 (75%) for chloramphenicol, and 4 (50%) for azithromycin. Regularly touched surfaces, such as hospital ward beds and door handles, are potential sources of nosocomial infections. These sites increase the contamination risk among susceptible hosts [23]. In the current investigation, 60 hospital ward beds and door handles were swabbed, and bacterial pathogens including *S. aureus, C. perfringes, K. pneumoniae, Clostridium spp*., and *Bacillus spp*. were detected.

In the current investigation, 60 hospital ward beds and door handles were swabbed, and bacterial pathogens including *S. aureus, C. perfringens, K. pneumoniae, Clostridium spp*., and *Bacillus spp*. were detected. Also, *C. perfringens* and *Clostridium spp*. were relatively new bacterial isolates from door handles and beds in hospital wards reported in the current study. This finding suggests poor hygienic practices by patients or health professionals before and after visiting the hospital toilets. *C. perfringens* is a spore-forming bacterium that is widely distributed in the environment and frequently occurs in the intestines of humans. Moreover, the spores of the bacterium are found in areas subject to human fecal pollution. Our result is in support of studies by Saadi and colleagues in Algeria and Tagoe *et al*. in Ghana, which reported *S. aureus, K. pneumoniae*, and *Bacillus spp*., among other bacterial species, isolated from patient beds and door handles in hospital wards [14,24]. Furthermore, similar bacterial isolates were detected on beds and door handles in Morocco and Nigeria [16,25]. On the contrary, our evidence is inconsistent with studies conducted in Ethiopia and Ukraine, which reported *S. aureus* as the only similar bacterial isolate [11,26]. Also, a contradictory finding was reported by a study in Cameroon, which showed that *K. pneumoniae, E. cloacae*, and *P. aeruginosa* are dominant bacterial contaminants on beds and door handles of hospital wards [27]. This disparity may be due to different sampling times, sampling techniques, culture methodologies, and hospital wards.

*S. aureus* constitutes part of the normal human flora, inhabiting the skin and mucous membranes, and is regularly shed into the hospital environment by patients and medical personnel. Taken together, *S. aureus* 21 (35%) was the most dominant bacterial contaminant in the current study. Staphylococci pathogens are dominant on surfaces because they can express adhesion factors and fatty acid-modifying enzymes for biofilm formation and modify anti-bacterial lipids, thereby increasing their survival for months on arid surfaces [14]. The elevated prevalence of *S. aureus* might be due to its resistance to the dry conditions of the hospital environment and transmission from the skin, nostrils, and boils of healthcare workers and patients [11]. Our report is following results from Algeria, Southwest Ethiopia, and Morocco [14-16]. However, Firesbhat *et al*. in Northwest Ethiopia and Barma and co-investigators in Nigeria found CoNS to be the most dominant bacterial contaminant on beds and door handles in wards [13,25]. Additionally, Ebongue *et al*., at a reference hospital in Douala, Cameroon, interestingly found *E. cloacae* as the predominant bacterial pathogen [27]. This variation could be due to differences in the hygienic conditions of study participants, the ventilation system, the frequency of surface decontamination, and the disinfection technique.

We most frequently detected *S. aureus* 17 (42.5%) from beds of hospital wards, and this finding is consistent with reports from Algeria, Southwest Ethiopia, Addis Ababa in Ethiopia, Southern Ethiopia, and Morocco [11,14-16,28]. Conversely, our evidence does not tie in with studies conducted in Northwest Ethiopia, Hawassa University Comprehensive Specialized Hospital of Ethiopia, a Douala reference hospital in Cameroon, and the University of Maiduguri Teaching Hospital in Nigeria [13,17,25,27]. *C. perfringens*7 (35%) was the most dominant bacterial contaminant on door handles in this study. However, contradictory results have been reported by studies carried out in Nigeria, Cameroon, Northwest Ethiopia, Algeria, Southwest Ethiopia, Southern Ethiopia, and Morocco [11,13-16,25,27]. Moreover, the disparity in results could be due to undercooking of food, wrong food storage, and poor hygienic practices (before and after every toilet visit) of health personnel and patients in this study.

The majority of the isolates of *S. aureus* in our study was susceptible to gentamycin 20 (95.2%) and azithromycin 20 (95.2%), followed by ampicillin 9 (42.9%), and chloramphenicol 8 (38.1%). On the other hand, Worku *et al*. in Southwest Ethiopia discovered that 11 (57.8%) of *S. aureus* was sensitive to ciprofloxacin, 6 (31.6%) to gentamycin, 5 (26.3%) to cefoxitin, and 5 (26.3%) to amoxicillin [15]. Also, Firesbhat *et al*. study in Northwest Ethiopia showed that ciprofloxacin was the most active (9 (60%)) agent against *S. aureus*, followed by cotrimoxazole 8 (53.3%) and gentamycin 8 (53.3%) [13]. Sebre *et al*. at the Tikur Anbessa Specialized Teaching Hospital in Addis Ababa, Ethiopia, reported *S. aureus* sensitivity rates of 55 (88.7%), 53 (84.1%), and 51 (81%) for clindamycin, gentamycin, and ciprofloxacin, respectively [28]. This difference in antimicrobial susceptibility among bacteria might be due to different general mechanisms of resistance, notably the study setting.

*C. perfringes* were most sensitive to gentamycin 6 (75%) and chloramphenicol 6 (75%). Gentamycin creates fissures in the outer membranes of gram-negative aerobic bacteria, leading to cell leakage and energy-mediated gentamycin uptake [29]. Therefore, gentamycin is less active against gram-positive anaerobes, including *C. perfringes*. Nevertheless, gentamycin was highly active against *C. perfringes* in this investigation. This may be because of the enhanced sensitivity (collateral sensitivity) of *C. perfringes* to gentamycin due to mutations causing multidrug resistance in *C. perfringes* [30]. Our research is nearly in agreement with an Iranian study performed among patients and healthy individuals at the Imam Reza Educational and Medical Center, which found that ceftriaxone 77 (97.46%) and chloramphenicol 75 (94.93%) were the most active antimicrobials against *C. perfringes* isolated from stool samples of diarrhea and non-diarrhea patients [31]. Our result is also nearly similar to another study conducted in South Africa, which showed that *C. perfringes* isolated from patients with suspected mixed aerobic/anaerobic infections were highly sensitive to chloramphenicol (100%), ertapenem (97.2%), and piperacillin-tazobactam (99.4%) [32]. Camacho *et al*. in Costa Rica reported a corroborating finding that demonstrated that cefotaxime, chloramphenicol, and imipenem were highly active agents against *C. perfringes* isolated from fecal samples of patients diagnosed with antibiotic-associated diarrhea [33].

Studies conducted in France, Southeastern Hungary, and Iraq have revealed contrary antibiotic sensitivity rates: (tigecyclin 86.5% and clindamycin 59.5%), (amoxicillin/clavulanic acid 100%, cefoxitin 100%, meropenem 100%, imipenem 100%, and metronidazole 100%), and (penicllins and β-lactamase inhibitors, metronidazole, and aminoglycosides), respectively, of *C. perfringes* isolated from wounds of patients or fecal samples of neonates [34-36]. These discrepancies in antibiotic-sensitivity profiles might be attributed to inappropriate administration of antimicrobials or variations in hospital environmental conditions, self-medication practices, and personal hygiene.

To our knowledge, this is the first study that has reported *C. perfringes* and *Clostridium spp*. from beds and door handles in hospital wards. Secondly, we found that *C. perfringes* is the most dominant bacterial pathogen on ward door handles. Thirdly, gentamycin was identified as an active agent against *C. perfringes*, in addition to chloramphenicol, which is an already-established antibiotic against *C. perfringes*. Lastly, *C. perfringes* was resistant to cephalothin.

**Study limitations:** the number of hospitals included in this study was small, which led to a limited sample size. Moreover, only seven different antibiotic discs were used to determine the antibiotic susceptibility of bacterial isolates. Therefore, the generalizability of our findings may be affected. Furthermore, *Clostridium spp*. other than *C. perfringes* could not be isolated in this study because of limited laboratory resources for laboratory analysis.

## Conclusion

*S. aureus* and *Bacillus spp*. had the highest contamination rates on beds and door handles in hospital wards. *C. perfringes* is the most common bacterial contaminant on door handles, while *S. aureus* is most commonly isolated from patient beds. *S. aureus, K. pneumoniae, Bacillus spp*., and *Clostridium spp*. showed the highest sensitivity to gentamycin. These isolates, except *K. pneumoniae*, were multi-drug resistant and most resistant to bacitracin. Food safety measures should be strictly followed in hospitals to prevent or manage outbreaks of foodborne diseases. Hospital beds and door handles should be regularly and adequately disinfected and cleaned. Hand hygiene should be routinely practiced by patients and healthcare workers through hand washing or the provision and use of hand sanitizers.

### What is known about this topic

- S. aureus, P. aeruginosa, Enterobacter, CoNS, Staphylococcus saprophticus, Klebsiella spp., CoNS, and Acinetobacter baumannii are found on beds and door handles of hospital wards;
- S. aureus is most dominant on patient beds in hospital wards;
- S. aureus is sensitive to gentamycin, ampicillin, and ciprofloxacin.

### What this study adds

- Clostridium perfringens sand Clostridium spp. also colonize wards’ beds and door handles;
- Door handles in hospital wards are most frequently contaminated with C. perfringens;
- Gentamycin is an active agent against C. perfringens.
